# Effectiveness of Telemonitoring in Obstetrics: Scoping Review

**DOI:** 10.2196/jmir.7266

**Published:** 2017-09-27

**Authors:** Dorien Lanssens, Thijs Vandenberk, Inge M Thijs, Lars Grieten, Wilfried Gyselaers

**Affiliations:** ^1^ Mobile Health Unit Facultiy of Medicine and Life Sciences Hasselt University Hasselt Belgium; ^2^ Department of Gynaecology Ziekenhuis Oost-Limburg Genk Belgium; ^3^ Department of Physiology Hasselt University Hasselt Belgium

**Keywords:** review, telemonitoring, obstetrics, maternal outcomes, fetal outcomes

## Abstract

**Background:**

Despite reported positive results of telemonitoring effectiveness in various health care domains, this new technology is rarely used in prenatal care. A few isolated investigations were performed in the past years but with conflicting results.

**Objective:**

The aim of this review was to (1) assess whether telemonitoring adds any substantial benefit to this patient population and (2) identify research gaps in this area to suggest goals for future research.

**Methods:**

This review includes studies exploring the effectiveness of telemonitoring interventions for pregnant women reported in the English language. Due to the paucity of research in this area, all reports including uncontrolled nonrandomized and randomized controlled studies were selected.

**Results:**

Fourteen studies, which performed their data collection from 1988 to 2010, met the inclusion criteria and were published from 1995 to present; four of the 14 published papers were multicenter randomized controlled trials (RCTs), five papers were single-center RCTs, three papers were retrospective studies, one paper was an observational study, and one paper was a qualitative study. Of the 14 papers, nine were available for a risk of bias assessment: three papers were classified as *low risk*, one as *medium risk*, and five as *high risk*. Furthermore, of those 14 papers, 13 focused on telemonitoring for maternal outcomes, and nine of the 14 papers focused on telemonitoring for fetal or neonatal outcomes. The studies reviewed report that telemonitoring can contribute to significant reductions in health care costs, (unscheduled) face-to-face visits, low neonatal birth weight, and admissions to the neonatal intensive care unit (NICU), as well as prolonged gestational age and improved feelings of maternal satisfaction when compared with a control group. When only studies with low risk of bias were taken into account, the added value of telemonitoring became less pronounced: the only added value of telemonitoring is for pregnant women who transmitted their uterine activity by telecommunication. They had significant prolonged pregnancy survivals, and the newborns were less likely to be of low birth weight or to be admitted to the NICU. Following these results, telemonitoring can only be recommended by pregnant women at risk for preterm delivery. It is however important to consider that these studies were published in the mid-90s, which limits their direct applicability given the current technologies and practice.

**Conclusions:**

This review shows that telemonitoring can be tentatively recommended for pregnant women at risk for preterm delivery. More recent RCTs with a blinded protocol are needed to strengthen the level of evidence around this topic and to have an insight in the added value of the technologies that are available nowadays. In addition, studies investigating patient satisfaction and economic effects in relation to telemonitoring are suggested for future research.

## Introduction

With more than 6 billion mobile phone subscribers worldwide, it is estimated that 75% of the world population has access to mobile communication. The number of devices with broadband capabilities has increased to more than 1 billion worldwide [1]. With more than 97,000 health-related mobile apps available and approximately 1000 new apps published every month, the potential to perform telemedicine exists [1]. Telemedicine is a relatively new approach (dating back to the early 1990s), which facilitates patients’ management at home [2]. It can be broadly defined as the use of telecommunication technologies to assist in the transmission of medical information and services between health care providers and patients. The use of this two-way telecommunication technology, multimedia, and computer networks to deliver or enhance the delivery of health care is a growing trend internationally [3]. It has the potential to improve access to high-quality disease management, and telemonitoring, a subgroup of telemedicine, has developed rapidly over the past decade [4]. There are several types of telemonitoring, ranging from simple to complex. In the simplest model, a patient receives support from a health care professional over the telephone. The patient monitors his or her symptoms and reports this during a structured telephone call. Moving up the scale of complexity is patient-initiated electronic monitoring with the transfer of physiologic data and record of symptoms by telephone or a broadband Internet connection from the patient’s home (ie, home telemonitoring) to the health care professional. On reviewing the data, the health care professional can contact the patient to request further information before making a decision about disease management. Finally, implanted monitoring devices transmit data wirelessly from the patient to a unit that is connected to a telephone or the Internet. Once again, if the data raise concern, the health care professional can contact the patient to request further information before making a decision about care [4].

A number of systematic reviews have evaluated the effectiveness of telemonitoring interventions for patients diagnosed with chronic cardiovascular disease (CVD), chronic obstructive pulmonary disease (COPD), and diabetes [2,5-8]. These reviews show mainly positive results and suggest that there is tentative evidence that telemonitoring may offer clinical benefit in these three domains. All-cause mortality and heart-related hospitalizations are reduced for patients with CVD compared with patients who received usual care [5,6]. Even primary care management of CVD can be enhanced by improving patient outcomes and reducing health-related costs [7]. Web-based remote monitoring for managing type 2 diabetes mellitus is also a viable approach for health care delivery and enhances patients’ quality of life [8]. Finally, home telemonitoring in patients with COPD appears to have a positive effect in reducing respiratory exacerbations and hospitalizations and in improving quality of life: patients with COPD were generally satisfied with home telemonitoring and found the systems useful to help them manage their disease and improve health care provision [2,9]. With regard to fertility, a few papers on telemonitoring discussing self-operated endovaginal telemonitoring of the ovarian stimulation phase in in vitro fertilization or intracytoplasmic sperm injection are published. This technique leads to relevant clinical decisions; significantly higher satisfaction of patients and their partner; a higher feeling of empowerment, discretion, and more active partner participation; as well as a trend toward less stress versus a traditional monitored group [10-12]. Despite the mainly positive results in the various health care domains and the ability to perform telemonitoring because of the improvement of technology, telemonitoring is rarely used in prenatal care. A few independent investigations were performed in the last years, but a systematic review has not yet been accomplished. For this reason, a systematic review of all clinical trials evaluating telemonitoring in high-risk pregnancies was performed. First, the characteristics of the study will be described, and then the maternal and neonatal outcomes in telemonitoring group versus control group (CG) will be reported. We aim to (1) assess whether telemonitoring adds any substantial benefit in the pregnant women population and (2) identify research gaps in this area and thereby suggest topics for future research.

## Methods

### Search Strategy

The following databases were comprehensively searched in August 2016 by two independent researches: the Medical Literature Analysis and Retrieval System Online (MEDLINE), the Cumulative Index to Nursing and Allied Health Literature (CINAHL), Excerpta Medica Database (EMBASE), PubMed, Limo, and the Cochrane Library. The enumeration of selected relevant journals was manually screened, and the bibliographies of all retained papers were examined for relevant studies. A third reviewer resolved discrepancies in judgment and verified the completeness of the manuscript.

### Search Items

The following terms were used in the search bar of the mentioned databases: “remote monitoring,” “telemonitoring,” “home monitoring,” “telemedicine,” “maternal health,” “telehealth,” “e-health,” “pregnancy,” “pregnancy-outcomes,” “gynecology,” “gravidity,” and “obstetrics.” Also medical subject headings (MeSH) thesaurus combined were used with the following terms: “blood pressure monitoring, ambulatory,” “blood glucose self-monitoring,” “pregnancy,” pregnancy outcome,” “gynecology,” and “obstetrics.”

### A Definition of Telemonitoring

In this review, we specified the definition of telemonitoring—as stated in the introduction—further to the following inclusion criteria: (1) require the patient to periodically measure physiological parameters (eg, blood pressure and weight) and/or record their symptoms or vital signs in a standardized format, (2) use telecommunication technologies (eg, mobile phone and Internet) that either manually or automatically transferred the patient’s health status data from home to a health care service, and (3) lead to the automated or manual review of the patient’s health status data.

**Figure 1:**
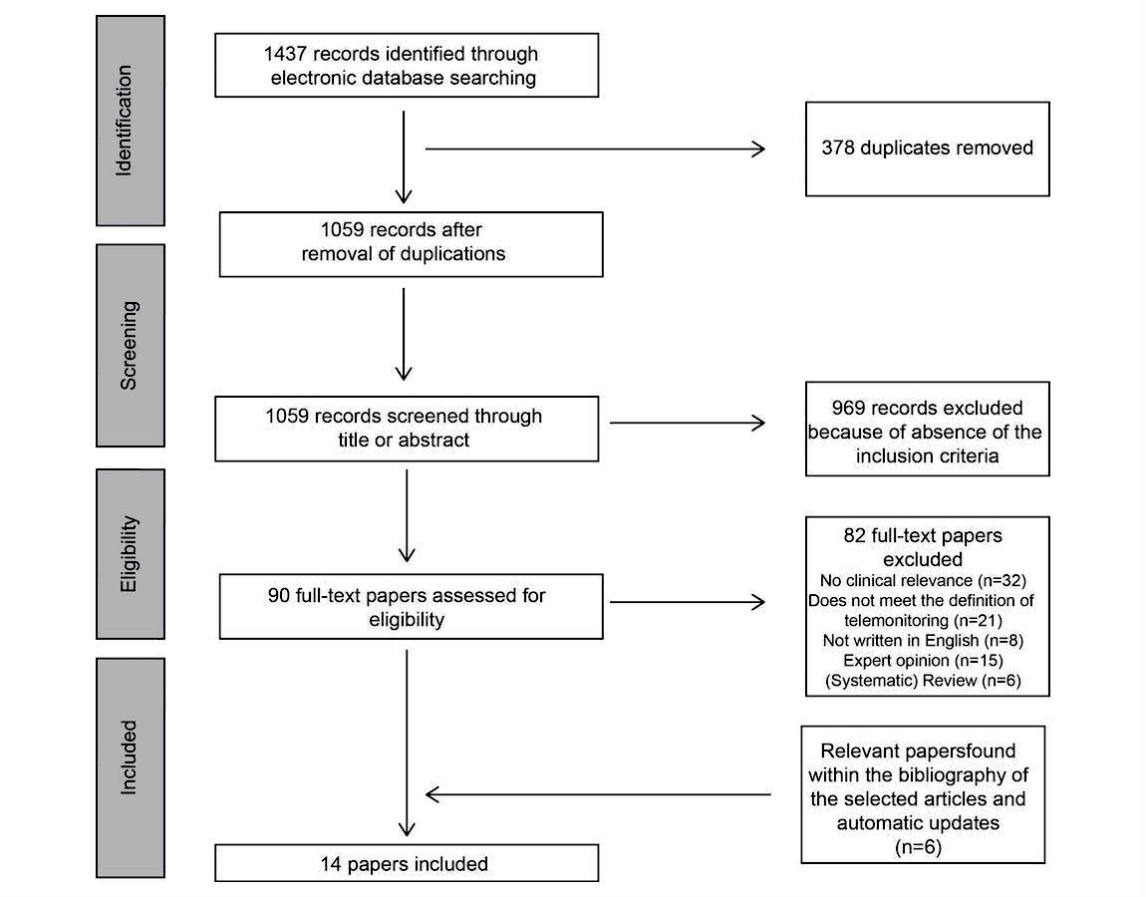
Selection procedure.

### Inclusion and Exclusion Criteria

To be included, studies had to examine the effectiveness of telemonitoring interventions for pregnant women as defined above. Scoping searches indicated a paucity of research in this area, and we therefore included uncontrolled and nonrandomized, as well as randomized controlled studies. All published studies reporting economic and/or clinically related outcomes (eg, hospital admission and preterm labor) were considered. Due to the scarce available publications, no time limitations were applied. All papers had to be written in English. Studies were excluded if health care professionals conducted the measurement of physiological signs at the patient’s home. In addition, review papers, expert opinions, and single case or case series reports were excluded.

### Selection Procedure

A flowchart of the selection procedure is shown in Figure 1. The database search identified 1437 papers. After the removal of duplicates, 1059 records were screened for relevant content. During title, abstract, and keyword screening, 969 papers were excluded because of the absence of the inclusion criteria. The full-text of the 90 potentially relevant papers was assessed, and 82 papers were excluded. Reasons for exclusion included (1) no clinical or economical relevance (n=32), (2) does not meet the definition of telemonitoring (n=21), (3) not written in English (n=8), (4) expert opinions (n=15), and (5) (systematic) reviews (n=6). Automatic updates from the databases and search for relevant papers within the bibliography of selected papers retrieved six papers, which were also included. In total, 14 papers were included.

### Assessment of Risk of Bias in Included Studies

A report on the methodological risk of bias of included studies (which had a randomized controlled design) in accordance with the Cochrane Handbook for Systematic Reviews of Interventions [13] and the guidelines of the Cochrane Consumers and Communication Review Group was made (Multimedia Appendix 1). These guidelines recommend the explicit reporting of the following individual elements for randomized controlled trials (RCTs): random sequence generation, allocation sequence concealment, blinding (participants and personnel), blinding (outcome assessment), completeness of outcome data, and selective outcome reporting. Each item is judged as being at high, low, or unclear risk of bias as set out in the criteria provided by Higgins et al (2011). Studies will be deemed to be at the highest risk of bias if they are scored as at high or unclear risk of bias for either the sequence generation or allocation concealment domains, based on growing empirical evidence that these factors are particularly important potential sources of bias [13].

### Data Extraction

The following information was collected and tabulated from the included studies: description of patient population, sample size, whether any economic evaluation was performed, the nature of the intervention, and the outcomes reported.

## Results

### Study Characteristics

**Figure 2:**
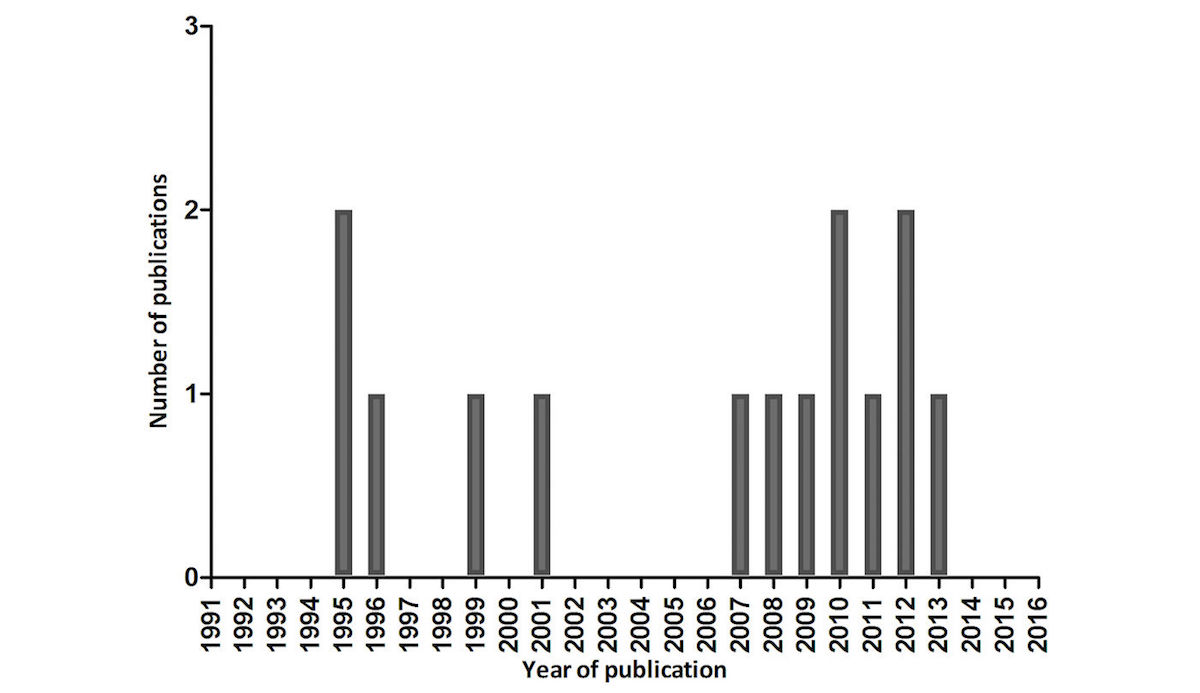
Number of publications during the last 25 years.

Fourteen studies were included, published from 1995 to present. An overview of these publication dates is presented in Figure 2.

Although the dates of the publications were from 1995 to present, the data collection was performed from 1988 to 2010.

Four of the 14 published papers were multicenter RCTs [14-17], five papers were single-center RCTs [18-22], three papers were retrospective studies [23-25], one paper was an observational study [26], and one paper was a qualitative study [27].

Multimedia Appendices 2 and 3 provide an overview of the characteristics of each study. All 14 papers report telemonitoring in obstetrics; 13 of the 14 papers focused on telemonitoring for maternal outcomes [14,15,17-27], and nine of the 14 papers focused on telemonitoring for fetal or neonatal outcomes [14-21,24]. Samples included varied from 15 singleton pregnancies [27] to 1292 singleton pregnancies [15]. Nine of the 14 papers were available for a risk of bias assessment (Multimedia Appendix 1): three papers were classified as *low risk* [14,15,25], one as *medium risk* [22], and five as *high risk* [17-21]. Five of the 14 papers did not have an RCT design [16,23,24,26,27]. For this reason, there was no risk of bias assessment made for them.

Telemonitoring data were generally transmitted to a monitoring center on a regular basis. Patients’ compliance with data transmission was assessed in three studies [18,19,25] and ranged from a mean of 21.8 (standard deviation [SD] 16.9) sets of data [18] to a mean of 35.6 (SD 32.3) sets of data [19], depending on the physiological parameter measured. All the data were automatically transferred in the studies that investigated the added value of telemonitoring in pregnancies at high risk for preterm delivery or with an induction [14-16,23-26]. The data of the studies which investigated the added value of telemonitoring in gestational diabetes mellitus (GDM) were manually transferred [17-21]. In almost all the studies, patients’ recordings outside predetermined values triggered an immediate action. Usual care included the same health care component as provided to the telemonitoring group but without telemonitoring.

### Maternal Outcomes

Multimedia Appendix 2 provides a summary of the 12 studies included focusing on telemonitoring for maternal outcomes: cervical dilatation or preterm labor, GDM, maternal satisfaction, and health care–related costs. These results will be further discussed below.

#### Cervical Dilatation or Preterm Labor

The use of telemonitoring in the monitoring of fetal heart rate and uterine activity dates back to the 1970s. The expected benefits lie in the prevention of perinatal mortality and morbidity [3]. In five studies, women with singleton pregnancies at high risk for preterm birth were randomly assigned to a telemonitoring group and a CG. The results of these studies are presented in Table 1.

#### Gestational Diabetes Mellitus

The application of telemedicine in the management of GDM has primarily focused on the transfer of blood glucose values from the patient to the provider, thereby eliminating frequent clinical visits and adverse maternal and fetal or neonatal outcomes [3]. Five studies did report these study outcomes (Table 2).

#### Maternal Satisfaction

Due to the new aspect of telemonitoring, the maternal satisfaction of these domain is rarely investigated. Table 3 summarizes the major findings of five studies after adding telemonitoring to the obstetrical care.

#### Health Care–Related Costs

The continuous strain on hospital bed occupancy puts clinicians under great pressure to discharge patients as soon as possible. It is assumed that telemonitoring can contribute to solve this problem. Two studies did compute these costs for a telemonitoring group in comparison with a CG (Table 4).

### Fetal or Neonatal Outcomes

Multimedia Appendix 3 provides a summary of the eight included studies focusing on telemonitoring for fetal or neonatal outcomes. In the next section, the influence of telemonitoring on the following fetal or neonatal outcomes will be presented: birth weight, gestational age, and submission to the NICU.

#### Birth Weight

Infants born small for gestational age (generally defined as less than 10th percentile) or large for gestational age (generally defined greater than 90th percentile) are at higher risk of short- and long-term morbidities than infants appropriately grown for gestational age [28]. A total of eight studies examined the impact of telemonitoring interventions on the birth weight of the neonate, which are presented in Table 5.

**Table 1:** Cervical dilatation or preterm labor and telemonitoring.

Citation	Risk of bias	Prolonged pregnancy survival	*P* value, TM^a^ group versus CG^b^	Experience of a preterm delivery	*P* value, TM group versus CG
Brown et al (1999) [22]	Low risk			−^c^	.73
Corwin et al (1996) [14]	Low risk	+^d^	.02	−	.04
CHUMS^e^ group (1995) [20]	Low risk			−	No^f,g^
Wapner et al (1995) [25]	Low risk	+	.016		
Morrison et al (2001) [24]	/^h^			−	<.001

^a^TM: telemonitoring.

^b^CG: control group.

^c^− indicates less experience in telemonitoring group versus control group.

^d^+ indicates more experience in telemonitoring group versus control group.

^e^CHUMS: Collaborative Home Uterine Monitoring Study.

^f^NS: not significant.

^g^No exact value is given.

^h^The slash indicates there was no risk of bias assessment possible for the study because of study design (eg, observational study).

**Table 2:** Gestational diabetes mellitus and telemonitoring.

Citation	Risk of bias	Fasting blood sugar	*P* value, TM^a^ group versus CG^b^	Glycated hemoglobin (HbA1c) <5.8%	*P* value, TM group versus CG	Insulin therapy	*P* value, TM group versus CG	Outpatient clinic visits	*P* value, TM group versus CG
Homko et al (2007) [18]	High risk	0^c^	No^d,e^			+^f^	<.05		
Dalfrà et al (2009) [17]	High risk			0	No	0	No^h^		
Homko et al (2012) [19]	High risk	0	.26			+	^e^		
Pérez-Ferre et al (2010) [20]	High risk							−^g^	<.001
Pérez-Ferre et al (2010) [21]	High risk			0	No			−	<.001

^a^TM: telemonitoring.

^b^CG: control group.

^c^0: no differences.

^d^NS: not significant.

^e^no exact value is given.

^f^+ indicates more experience in telemonitoring group versus CG.

^g^− indicates less experience in telemonitoring group versus CG.

**Table 3:** Maternal satisfaction and telemonitoring.

Citation	Risk of bias	Result for women in telemonitoring group
Homko et al (2007) [18]	High risk	More feelings of self-efficacy in women with GDM^a^
Dalfrà et al (2009) [17]	High risk	Women in the telemonitoring group showed lower levels of frustration and concerns about their GDM and a better acceptance of their diabetic condition
O’Brien et al (2013) [27]	/^b^	Better birth experiences resulting by induction of labor at home
Pérez-Ferre et al (2010) [21]	High risk	Higher patient satisfaction in women with GDM
Rauf et al (2011) [26]	/	Labor induction at home is feasible and acceptable to women

^a^GDM: gestational diabetes mellitus.

^b^The slash indicates there was no risk of bias assessment possible for the study because of study design (eg, observational study).

**Table 4:** Health care–related costs and telemonitoring.

Citation	Risk of bias	Result for women in telemonitoring group versus women in control group	
		Total cost saving	Average cost saving per pregnancy
Buysse et al (2008) [23]	/^a^	€145,822 for 415 pregnant women	€351.38
Morrison et al (2001) [24]	/	US $867,540 for 60 pregnant women	US $14,459

^a^The slash indicates there was no risk of bias assessment possible for the study because of study design (eg, observational study).

**Table 5:** Birth weight and telemonitoring.

Citation	Risk of bias	Small for gestational age (<10th percentile)	*P* value, TM^a^ group versus CG^b^	Mean birth weight	*P* value, TM group versus CG	Large for gestational age (>90th percentile)	*P* value, TM group versus CG
CHUMS^c^ group (1995) [20]	Low risk	−^d^	No^e,f^	+^g^	No^e^		
Corwin et al (1996) [14]	Low risk	−	.003				
Homko et al (2007) [18]	High risk					+	No^e^
Dalfrà et al (2009) [17]	High risk			0^h^	No^e^	0	No^e^
Homko et al (2012) [19]	High risk			0	.30	+	.70
Morrison et al (2001) [24]	/^i^	−	.001	+	<.001		
Pérez-Ferre et al (2010) [20]	High risk			0	No^e^		
Pérez-Ferre et al (2010) [21]	High risk			0	.39	-	No^e^

^a^TM: telemonitoring.

^b^CG: control group.

^c^CHUMS: Collaborative Home Uterine Monitoring Study.

^d^− indicates less experiences or lower mean in telemonitoring group versus CG.

^e^NS: not significant.

^f^No exact value is given.

^g^+ indicates more experiences or higher mean in telemonitoring group versus CG.

^h^0= no differences.

^i^The slash indicates there was no risk of bias assessment possible for the study because of study design (eg, observational study).

**Table 6:** Gestational age and telemonitoring.

Citation	Risk of bias	<37 weeks	*P* value, TM^a^ group versus CG^b^	<36 weeks	*P* value, TM group versus CG	<35 weeks	*P* value, TM group versus CG	<34 weeks	*P* value, TM group versus CG	<32 weeks	*P* value, TM group versus CG
CHUMS^c^ group (1995) [20]	Low risk	+^d^	No^e,f^	−	No^e^			−	No^f^		
Homko et al (2007) [18]	High risk	0	No^e^								
Morrison et al (2001) [24]	/^g^					−	<.01			−	.003
Kuleva et al (2012) [16]	/			−	.016						

^a^TM: telemonitoring.

^b^CG: control group.

^c^CHUMS: Collaborative Home Uterine Monitoring Study.

^d^+ indicates more experiences or higher mean in telemonitoring group versus CG.

^e^NS: not significant.

^f^No exact value is given.

^g^The slash indicates there was no risk of bias assessment possible for the study because of study design (eg, observational study).

#### Gestational Age

We previously reported the influence of telemonitoring on cervical dilatation or preterm labor. One of the consequences of preterm labor is a preterm delivery of the newborn. Only four studies reported gestational age of the newborn as a main outcome. In Table 6, the rate of experiences of preterm births (for the gestational age of less than 37 weeks, less than 36 weeks, less than 35 weeks, less than 34 weeks, or less than 32 weeks) in telemonitoring group versus CG is reported.

#### Submission to Neonatal Intensive Care Unit (NICU)

Four studies have investigated the added value of telemonitoring and the submission to the NICU. These studies are presented in Table 7.

**Table 7:** Submission to the neonatal intensive care unit (NICU) and telemonitoring.

Citation	Risk of bias	Admission to NICU^a^	*P* value, telemonitoring group versus control group
CHUMS^b^ group (1995) [20]	Low risk	−^c^	No^d,e^
Corwin et al (1996) [14]	Low risk	−	.01
Homko et al (2007) [18]	High risk	+^f^	No
Morrison et al (2001) [24]	/^g^	−	<.001

^a^NICU: neonatal intensive care unit.

^b^CHUMS: Collaborative Home Uterine Monitoring Study.

^c^− indicates less experiences in telemonitoring group versus CG.

^d^NS: not significant.

^e^No exact value is given.

^f^+ indicates more experiences in telemonitoring group versus CG.

^g^The slash indicates there was no risk of bias assessment possible for the study because of study design (eg, observational study).

## Discussion

### The Low Level of Evidence Suggests a Potential Benefit of telemonitoring in Prenatal Care

This review provided a comprehensive description of the use of telemonitoring interventions in obstetrics. Nine of the 14 papers were published from 2007 to present, suggesting that telemonitoring interventions are a relatively new field in obstetrics research. The papers of telemonitoring, which included cervical dilatation or preterm labor as a main outcome, demonstrated that transmitting uterine activity by telecommunication resulted in significant prolonged pregnancy survivals [14,25]. The papers of telemonitoring for GDM demonstrated lower levels of frustration and concerns about their diabetes and a better acceptance of their diabetic condition [17], elated feelings of self-efficacy [18], and a reduction in (unscheduled) face-to-face visits [20,21] in the telemonitoring group compared with the CG. Additionally, a cost reduction [23,24] and elevated feelings of maternal satisfaction [18,26,27] were obtained when telemonitoring was used in obstetrical care. The newborns did have a higher gestational age at delivery [24] and were less likely to be of low birth weight [14,24] or to be admitted to the NICU [14,24] when a telemonitoring group was compared with a CG. Fetuses with abnormal versus normal fetal heart rate at home monitoring were more likely to have an earlier gestational age [16].

Despite the mainly positive results described above, a distinction between studies with low methodological risk of bias assessment and studies with high methodological risk of bias assessment has to be made. When only studies with low risk of bias assessment were taken into account, the added value of telemonitoring became less pronounced. Only pregnant women who transmitted their uterine activity by telecommunication would experience benefits of this technology. They had significant prolonged pregnancy survivals [14,25], and the newborns were less likely to be of low birth weight [14] or to be admitted to the NICU [14]. The study by the Collaborative Home Uterine Monitoring Study (CHUMS) group (1995) was rated low risk for bias but did not mention any significant results for these metrics. On the basis of the low risk for bias criteria, telemonitoring appears to be useful for reducing preterm delivery for pregnant women at risk, but caution should be exercised because only two high-quality studies reported that these benefits were found. Additionally, these papers with a low risk for bias were published in the mid-90s. Their conclusions are questionable when we want to adapt them to current practice because of rapid changes in technology.

### Research Gaps and Suggestions for Future Research

Despite the positive results, which are reported above, further research needs to be done to define the added value of telemonitoring and advocate the use of this intervention as a patient management approach in clinical practice. Three main recommendations for future research are made, based on the research gaps elucidated through this review:

1. The level of evidence of the included papers is not high. When a methodological risk of bias is performed, four of these studies classified as *high risk*, one as *medium risk*, and three as *low risk*. Information about randomization (random sequence generation and allocation of concealment) was often lacking; blinding of participants, personnel, and outcomes was not performed in most studies; and none of the used protocols in the intervention groups were available. The level of evidence of the other five studies (which were retrospective studies, a qualitative study, and an observational study) was much lower. There is a need for new multicentric RCTs on different pregnancy conditions in which a blinding for both the patients and the caregivers as the outcomes is performed but with well-considered decisions regarding the ethical aspects. This is to (1) associate the potential of telemonitoring interventions with maternal and fetal outcomes, (2) verify the results which were observed in the mentioned study, (3) investigate the added value of the new technologies nowadays, and (4) improve the evidence on this topic with rigorous research designs.
2. Only four studies reported maternal satisfaction in relation with the use of telemonitoring during their pregnancy (two of them about the use of telemonitoring in pregnancies complicated with GDM and two in the context of labor induction at home). These studies have a relatively small patient population, ranging from 15 to 70 pregnant women. Patients’ satisfaction with the use of telemonitoring systems should be further explored using more robust and validated instruments. Additionally, an evaluation of satisfaction of telemonitoring when used in pregnancies with other pregnancy complications (such as gestational hypertension and premature contractions) and in a bigger patient population is recommended. Alternatively, a thorough qualitative analysis can be conducted to enable an in-depth understanding of patients’ satisfaction and the use of that information to improve future technology designs. This may help adjusting the interventions to the target population and can have a positive impact on various domains such as patient compliance and birth experiences.
3. Only two studies performed a cost analysis of prenatal care, including telemonitoring. Both were retrospective studies that were not assessed for risk of bias. Although these studies demonstrated the possibility of cost reduction with the use of telemonitoring, there were visible shortcomings in the study designs. Buysse et al (2008) performed a retrospective study and did not include variables such as time-travel distance from home to hospital and the patient’s actual clinical condition. In addition, the staffing costs and equipment costs (based on a reasonable estimate) were not taken into account. Additionally, the data in the study of Morrison et al (2001) were retrospectively collected and did not include the actual clinical condition. In contrast to the previous mentioned study, they asked a fee to finance telemonitoring costs. It is challenging to examine the cost benefit of telemonitoring when it’s added to standard prenatal care and whether this is beneficial in both high- and low-risk pregnancies. We recently stated that new technologies can reduce the medicalization of prenatal care [29], but further studies with a prospective design and patient specific treatment(s) are needed to substantiate or reject this hypothesis and to evaluate the cost-effectiveness and health care utilization of telemonitoring in obstetrical care.

### Limitations

This review has several limitations that need to be acknowledged. First, the studies were restricted to the English language. Although records written in other languages were excluded, they could be relevant in the scope of this review. Second, a key limitation in the included papers is the heterogeneity of the interventions reported by the investigators. Telemonitoring interventions are frequently multidimensional, containing a range of elements, including the transmission of physiological data, coaching, telephone support, nurse interventions, and Web-based communications [8]. A few studies had a clearly stated aim for the telemonitoring intervention implemented, but, in general, the telemonitoring intervention is poorly described, especially in terms of the assessment of the data transferred and how this assessment leads to a service response or not. Third, the rapid technological advancements that have been seen in the last decade may also impact the ability to compare older and newer studies using different technology. The oldest study dates from 1995, the most recent from 2012. Finally, there was almost no information concerning missing data or the compliance of the patients. The often missing information about compliance rates suggests that telemonitoring regimens may not be appropriate for all patients.

### Conclusions

Overall, this review has shown the added value, for both mother and child, of telemonitoring used in a prenatal follow-up program in obstetrical care. However, most of the included studies have a high methodological risk of bias. When only studies with low risk of bias are taken into account, the added value of telemonitoring became less pronounced. Only the pregnant women who transmitted their uterine activity by telecommunication had significant benefits from this technology: they experienced prolonged pregnancy survivals, and the newborns were less likely to be of low birth weight or to be admitted to the NICU. On the basis of the limited results of two high-quality studies conducted in the mid-90s, telemonitoring can be tentatively recommended for pregnant women at risk for preterm delivery. However, more recent RCTs with a blinded protocol and studies investigating patient satisfaction and economic effects in relation to telemonitoring are suggested for future research.

## Appendix

Multimedia Appendix 1 Assessment of risk of bias in included studies.

Multimedia Appendix 2 Summary table of included studies – maternal outcomes.

Multimedia Appendix 3 Summary table of included studies – neonatal outcomes.

## Abbreviations

CG: control group
CHUMS: Collaborative Home Uterine Monitoring Study
COPD: chronic obstructive pulmonary disease
CVD: cardiovascular disease
GDM: gestational diabetes mellitus
NICU: neonatal intensive care unit
NS: not significant
RCT: randomized controlled trial
SD: standard deviation
TM: telemonitoring
