# Supplementary material for: Effectiveness of Telemonitoring in Obstetrics: Scoping Review
Source: J Med Internet Res. 2017 Sep 27;19(9):e327. doi: 10.2196/jmir.7266 (PMC5637065; doi:10.2196/jmir.7266)
Supplement: Multimedia Appendix 1 [file jmir_v19i9e327_app1.pdf]

Appendix 1: Assessment of risk of bias in included studies

**Article 1:** A multicenter randomized controlled trial of home uterine monitoring: Active versus sham device.<br>The collaborative Home Uterine Monitoring Study (CHUMS) Group (1995) [15]		
**Domain**	**Review authors' judgement**	**Support to judgement**
**Random sequence generation**	High risk<br>Unclear<br>Low risk	Computer generated randomization scheme provided by an off-site consultant firm. To ensure approximately equal allocation, blocked random number sequences were used.
**Allocation concealment**	High risk<br>Unclear<br>Low risk	The concealing was done by an off-site consultant firm. The computer-generated randomization scheme was used to assign patients consecutively without regard to specific risk factor.
**Blinding of participants and personnel**<br>Assessments should be made for each main outcome (or class of outcomes)	High risk<br>Unclear<br>Low risk	The identity of group assignment was blinded to patients and their caregivers through the completion of the entire study.
**Blinding of outcome assessment**<br>Assessments should be made for each main outcome (or class of outcomes)	High risk<br>Unclear<br>Low risk	Patients in each group received identical education regarding device use and transmission protocol, signs and symptoms of preterm labor, and self-palpation for uterine contractions (required during all monitoring sessions).<br>The uterine activity data of the active groups patients were electronically 'buried' and not seen by the nurses.<br>At the times of the first daily home uterine activity monitoring transmission, all patients received similar daily phone contact with the base station nurses, which included an uniform scripted interview containing a standard list of signs and symptoms of preterm labors. All subsequent patient interactions with base station nurses followed scripted protocols that were similar for both groups whether they involved remote monitoring, alerting of physicians, or referral to hospitals.
**Incomplete outcome data**<br>Assessments should be made for each main outcome (or class of outcomes)	High risk<br>Unclear<br>Low risk	1355 patients were enrolled<br>- 1292 were randomized<br>o 637 active device<br>o 655 sham device<br>- 1165 used home uterine activity monitoring devices<br>o 574 active device<br>o 591 sham device<br>- 842 completed the study<br>o 405 active device

<table>
<tr><td></td><td></td><td>o 437 sham device
- 321 discontinued study
o Withdrew voluntarily
▪ 99 active device
▪ 78 sham device
o Noncompliant
▪ 72 active device
▪ 75 sham device
o Unable to locate
▪ 5 active device
▪ 13 sham device
o Other medical reason
▪ 6 active device
▪ 9 sham device
o Other
▪ 39 active device
▪ 28 sham device
No re-inclusions were performed.</td></tr>
<tr><td>Selective reporting</td><td>High risk
Unclear
Low risk</td><td><u>Primary outcomes:</u> detection of preterm labour and reduction of preterm birth.
<u>Discussed outcomes:</u> change in cervical dilatation from previous visit at diagnoses of preterm labor, preterm labor diagnosed, cervical examination at diagnoses of preterm labor, incidence of PROM, gestational age at delivery, birth weight, admissions to neonatal care unit, length of hospitalization, neonatal complications</td></tr>
<tr><td colspan="3">Overall outcome: low risk of bias in this study</td></tr>
</table>

**Article 2:** A randomized multicenter trial assessing a home uterine activity monitoring device used in the absence of daily nursing contact.<br>Wapner et al. (1995) [25]		
**Domain**	**Review authors' judgement**	**Support to judgement**
**Random sequence generation**	High risk<br>Unclear<br>Low risk	Randomization and group assignments were carried out by study personnel not directly responsible for patient care. A separate blocked (n = 20) random number sequence was used at each site.
**Allocation concealment**	High risk<br>Unclear<br>Low risk	Randomization and group assignments were carried out by study personnel not directly responsible for patient care and were performed by opening consecutively numbered envelopes at each study site.
**Blinding of participants and personnel**<br>Assessments should be made for each main outcome (or class of outcomes)	High risk<br>Unclear<br>Low risk	Participants: no blinding was done. The unmonitored group received routine high-risk obstetric care without home uterine activity monitoring, whereas the monitored group received the same high-risk obstetric care with home uterine monitoring.<br>Personnel: primary care physicians were blinded to their patient's group assignment and all health care personnel participating in the judgment of preterm labor were instructed to inquire regarding the patient's group assignment.
**Blinding of outcome assessment**<br>Assessments should be made for each main outcome (or class of outcomes)	High risk<br>Unclear<br>Low risk	Primary care physicians were blinded to their patient's group assignment and all health care personnel participating in the judgment of preterm labor were instructed to inquire regarding the patient's group assignment.<br>If, during an office or hospital visit, the examining physician became aware of the patient's group assignment, another physician conducted a separate cervical examination for recording on the data collection form and determination of preterm labor. Patients were instructed that there was to be no mention of whether contractions were suspected on the basis of monitoring results or patients self-palpation.
**Incomplete outcome data**<br>Assessments should be made for each main outcome (or class of outcomes)	High risk<br>Unclear<br>Low risk	218 women were enrolled<br>- 187 women completed the study<br>o 107 in the monitored group<br>o 111 in the unmonitored group<br>- 29 were excluded<br>o 7 were withdrawn because the physician removed them or they later decided not to participate.

		o 3 were excluded because of pre-eclampsia or fetal distress before 37 weeks<br>o 4 were withdrawn because of the loss of telephone service<br>o 5 patients relocated and were lost to study follow-up<br>o 9 were enrolled into the monitor group but never received monitoring<br>o 1 had intrauterine fetal death before 37 weeks of gestation<br>- A total of 21 women in the monitored group and 22 in the unmonitored group completing the study experienced preterm labor.<br>No specific numbers of exclusion in the groups separately.<br>No re-inclusions were reported.
**Selective reporting**	High risk<br>Unclear<br>Low risk	Primary outcomes: cervical states as measured by cervical dilatation at the time of diagnoses of preterm labor.<br>Discussed outcomes: gestational age at diagnoses of preterm labor, cervical dilatation at diagnosis of preterm labor, change in cervical dilatation from prior examination, duration of gestation after diagnosis of preterm labor, delivery within 48 hours of detection of preterm labour.
Overall outcome: only the blinding of the participants was a high risk factor for bias in this study.		

**Article 3:** Multicenter randomized clinical trial of home uterine activity monitoring: Pregnancy outcomes for all women randomized.<br>Corwin et al. (1996) [14]		
**Domain**	**Review authors' judgement**	**Support to judgement**
**Random sequence generation**	High risk<br>Unclear<br>Low risk	All randomization and group assignment were performed by study personnel without direct patient care responsibilities.<br>No local study personnel had access to the group assignments.
**Allocation concealment**	High risk<br>Unclear<br>Low risk	After eligibility criteria had been met and informed consent was obtained, group assignment was made by means of opening consecutively numbered envelopes that randomized patients with a table of random numbers. A different random number sequence was used for each study site.
**Blinding of participants and personnel**<br>Assessments should be made for each main outcome (or class of outcomes)	High risk<br>Unclear<br>Low risk	Participants weren't blinded: the not-monitored group received standard high-risk obstetric care; the monitored group received the same standard high-risk obstetric care plus twice-daily home uterine activity monitoring.<br>Personnel was blinded: all patients were instructed not to inform caregivers of their group assignment. Caregivers were informed that they were seeing a study participant but not told the group assignment.
**Blinding of outcome assessment**<br>Assessments should be made for each main outcome (or class of outcomes)	High risk<br>Unclear<br>Low risk	Caregivers were informed that they were seeing a study participant but not told the group assignment. If uterine contractions were suspected, caregivers were not informed if the contractions were detected by the monitor or by the patient. The diagnosis of preterm labor for the purpose of this study required both more than four uterine contractions per hour and a change in cervical examination.
**Incomplete outcome data**<br>Assessments should be made for each main outcome (or class of outcomes)	High risk<br>Unclear<br>Low risk	Of the 339 women with singleton gestations (174 monitored and 165 not monitored) who enrolled in the study, 14 women (6 monitored, 8 not monitored), who moved during the study and were not available for follow-up data collection, are not included in the analyses of gestational age at delivery. Four women in the monitored group and three in the not-monitored group experienced fetal death

		before 24 weeks' gestation.<br>The remaining 164 monitored and 154 not monitored women had intact pregnancies at 24 weeks' gestation and had successful ascertainment of gestational age at delivery.<br>No specific numbers of exclusion in the groups separately.<br>No re-inclusions were reported.
**Selective reporting**	High risk<br>Unclear<br>Low risk	Primary outcomes: gestational age at the moment of delivery and preterm birth < 35 weeks of gestation.<br>Discussed outcomes:<br>- < 37 weeks of pregnancy<br>- < 31 weeks of pregnancy<br>- Birthweight < 2500 g<br>- Birthweight < 2000 g<br>- Birthweight < 1500 g<br>- Admission of neonatal intensive care unit
Overall outcome: only the blinding of the participants was a high risk factor for bias in this study.		

**Article 4:** A randomized comparison of home uterine activity monitoring in the outpatient management of women treated for preterm labour.<br>Brown et al. (1999) [22]		
**Domain**	**Review authors' judgement**	**Support to judgement**
**Random sequence generation**	High risk<br>Unclear<br>Low risk	?
**Allocation concealment**	High risk<br>Unclear<br>Low risk	The random assignment process used sealed opaque envelopes to determine whether a patient would be in the monitored of the control group.
**Blinding of participants and personnel**<br>Assessments should be made for each main outcome (or class of outcomes)	High risk<br>Unclear<br>Low risk	Patients weren't blinded: women randomly assigned to the monitored group were asked to transmit a uterine monitor strip by telephone to the monitor center twice daily until 37 weeks of gestation or until instructed to discontinue monitoring. women in the not-monitored group didn't receive a monitor.<br>Personnel wasn't blinded: the physician or perinatal nurse was notified by the monitor center if the patient exceeded the baseline contraction frequency of 6 contractions/h.
**Blinding of outcome assessment**<br>Assessments should be made for each main outcome (or class of outcomes)	High risk<br>Unclear<br>Low risk	?
**Incomplete outcome data**<br>Assessments should be made for each main outcome (or class of outcomes)	High risk<br>Unclear<br>Low risk	Of the 997 women screened for random assignment 34.4% (343/997) met study criteria. Among these 343 women 48 (14.0%) declined to participate, 81 (23.6%) had no telephone, 28 (8.2%) were released before consent could be obtained, and 186 (54.2%) were enrolled. Twenty-four women initially randomly assigned were ruled ineligible for various reasons. A total of 162 women were available for comparison:<br>- Monitored group: n = 82<br>- Control group: n = 80<br>No specific numbers of exclusion in the groups separately.<br>No re-inclusions were reported.
**Selective reporting**	High risk<br>Unclear<br>Low risk	Primary outcomes: evaluation of preterm birth < 35 weeks' gestation, readmissions for recurrent preterm labor and observations lasting < 24 hours, compliance.<br>Discussed outcomes:<br>- Delivery outcomes<br>o Gestation at delivery < 35 weeks<br>o Gestation at delivery 35 – 37 weeks

		o Gestation at delivery > 37 weeks<br>o Primary caesarean delivery<br>o Neonatal intensive care unit admissions<br>o Neonates receiving mechanical ventilation<br>o Antenatal corticosteroid treatment<br>- Neonatal outcomes:<br>o Gestational age at delivery<br>o Birth weight<br>o Neonatal intensive care unit stay<br>- Compliance with home uterine activity monitoring<br>o Gestation at delivery<br>▪ < 35 weeks<br>▪ ≥ 35 weeks<br>o Cervical dilatation at enrollment<br>▪ < 2 cm<br>▪ ≥ 2 cm<br>o Ethnicity<br>▪ African American<br>▪ White
Overall outcome: of the 6 domains were 2 unclear, 1 of high risk and 3 of low risk. The risk of bias is medium.		

**Article 5:** Use of an Internet-based telemedicine sytem to manage underserved women with gestational diabetes mellitus.<br>Homko et al. (2007) [18]		
**Domain**	**Review authors' judgement**	**Support to judgement**
**Random sequence generation**	High risk<br>Unclear<br>Low risk	?
**Allocation concealment**	High risk<br>Unclear<br>Low risk	?
**Blinding of participants and personnel**<br>Assessments should be made for each main outcome (or class of outcomes)	High risk<br>Unclear<br>Low risk	Patients and personnel weren't blinded: women in the treatment group were asked to transmit this information via the diabetes health network at least three times a week to their health care providers. Women in the control group were asked to record this information in a logbook, which was reviewed by the medical team at prenatal visits.
**Blinding of outcome assessment**<br>Assessments should be made for each main outcome (or class of outcomes)	High risk<br>Unclear<br>Low risk	All data were analyzed based on subjects' assigned group.
**Incomplete outcome data**<br>Assessments should be made for each main outcome (or class of outcomes)	High risk<br>Unclear<br>Low risk	63 women with gestational diabetes consented to participate in the study protocol and were randomized to either the telemedicine group or the control group:<br>- 34 women were randomized to the intervention group<br>o 2 women withdrew from the study<br>- 29 women were randomized to the control group<br>o 2 women were loss to follow-up<br>o 1 woman was excluded because of a twin gestation<br>o 1 woman did not met the inclusion criteria<br>No re-inclusions were reported.
**Selective reporting**	High risk<br>Unclear<br>Low risk	Primary outcomes: sytem use, maternal feelings of diabetes self-efficacy.<br>Secondary outcomes: maternal glucose control and pregnancy outcomes (mode of delivery, gestational age at delivery, infant birth weight, length, neonatal intensive care unit admission, Apgar scores, rates of large for gestational age and other neonatal morbidities.<br>Discussed outcomes:<br>- diabetes therapy maternal feelings of diabetes self-efficacy<br>- maternal glucose control<br>- maternal outcomes:<br>o cesarean delivery

		o pre-eclampsie/gestational hypertension<br>o premature rupture of membranes<br>o placental abruption<br>- neonatal outcomes:<br>o birthweight<br>o large for gestational age<br>o gestational age at delivery<br>o Apgar score<br>o Intensive care unit admissions<br>o Preterm delivery<br>o Composite outcome<br>o Neonatal hypoglycemia<br>o Jaundice/hyperbilirubinemia<br>o Respiratory distress syndrome
Overall outcome: of the 6 domains were 4 of high risk and 2 of low risk. The risk of bias is high.		

**Article 6:** The effect of telemedicine on outcome and quality of life in pregnant women with diabetes.<br>Dalfrà et al. (2009) [17]		
**Domain**	**Review authors' judgement**	**Support to judgement**
**Random sequence generation**	High risk<br>Unclear<br>Low risk	?
**Allocation concealment**	High risk<br>Unclear<br>Low risk	?
**Blinding of participants and personnel**<br>Assessments should be made for each main outcome (or class of outcomes)	High risk<br>Unclear<br>Low risk	Participants: no blinding was done. Women in the telemedicine groups were given training on the use of the equipment and they were asked to submit their data every week, while they had a medical examination at the diabetes clinic every month. Women in the control groups had a medical examination every two week.<br>Personnel: not mentioned if they were blinded. A diabetes team evaluated the patients data.
**Blinding of outcome assessment**<br>Assessments should be made for each main outcome (or class of outcomes)	High risk<br>Unclear<br>Low risk	?
**Incomplete outcome data**<br>Assessments should be made for each main outcome (or class of outcomes)	High risk<br>Unclear<br>Low risk	276 pregnant women were enrolled in the study<br>- 240 did have gestational diabetes<br>o 7 were excluded because they did not complete the questionnaires at the end of the study<br>o 88 were in the telemedicine group<br>o 115 in the control group<br>- 36 did have diabetes type 1<br>o 4 were excluded because they did not complete the questionnaires at the end of the study<br>o 17 were in the telemedicine group<br>o 15 in the control group<br>No re-inclusions were reported.
**Selective reporting**	High risk<br>Unclear<br>Low risk	Primary outcomes: pre-pregnancy BMI, week of gestation when diabetes was diagnosed, duration of diabetes, therapy, HbA1c at enrollment and at the end of the pregnancy, timing and mode of delivery, maternal

		complications, newborn's weight, presence of macrosomia and complication, depression, health-related quality of life, stress and distress for the impact of diabetes.<br>Discussed outcomes: pre-pregnancy BMI, week of gestation when diabetes was diagnosed, duration of diabetes, therapy, HbA1c at enrollment and at the end of the pregnancy, timing and mode of delivery, maternal complications, newborn's weight, presence of macrosomia and complication, depression, health-related quality of life, stress and distress for the impact of diabetes.
Overall outcome: of the 6 domains were 4 of high risk and 2 of low risk. The risk of bias is high.		

**Article 7:** The outcomes of gestational diabetes mellitus after a telecare approach are not inferior to tradition outpatient clinic visits.<br>Pérez-Ferre et al. (2010) [20]		
**Domain**	**Review authors' judgement**	**Support to judgement**
**Random sequence generation**	High risk<br>Unclear<br>Low risk	?
**Allocation concealment**	High risk<br>Unclear<br>Low risk	?
**Blinding of participants and personnel**<br>Assessments should be made for each main outcome (or class of outcomes)	High risk<br>Unclear<br>Low risk	Patients and personnel weren't blinded: patients in the monitored group need to send blood glucose values recorded in the glucometer to the medical terminal once a week; patients in the not-monitored group received standard care. An endocrinologist and a diabetes nurse educator evaluated patients' data accessing to a web application from any PC with Internet Connection. They had access to blood glucose values sent by the patients, accompanied by their identification by patients' signals, date and time of measurement.
**Blinding of outcome assessment**<br>Assessments should be made for each main outcome (or class of outcomes)	High risk<br>Unclear<br>Low risk	?
**Incomplete outcome data**<br>Assessments should be made for each main outcome (or class of outcomes)	High risk<br>Unclear<br>Low risk	100 women gave their written informed consent and were allocated to:<br>- The intervention group (n = 50)<br>o 48 women completed the study<br>o 2 women were excluded; reason?<br>- The control group (n = 50)<br>o 49 women completed the study<br>o 1 woman was excluded; reason?<br>No re-inclusions were reported.
**Selective reporting**	High risk<br>Unclear<br>Low risk	Primary outcomes: pregnancy, delivery and newborn data and outcomes of women with gestational diabetes mellitus when treated using a fluid telemedicine system as compared with standard outpatient clinical care.<br>Discussed outcomes:<br>- Gestational age at delivery<br>- Pregnancy induces hypertension<br>- Normal vaginal birth<br>- Dystocia<br>- New born gender<br>- Birth weight

		- Large for gestational age<br>- Hypoglycemia<br>- Hypokaliemia<br>- Hypocalcemia<br>- Poliglobulia<br>- Small for gestational age<br>- Preterm birth<br>- Loss of fetal wellbeing<br>- Umbilical cord pathology<br>- Shoulders dystocia<br>- Abruptio placentae
Overall outcome: of the 6 domains were 4 of high risk 0, 1 was unclear and 1 of low risk. The risk of bias is high.		

**Article 8:** A telemedicine system based on internet and short message service as a new approach in the follow-up of patients with gestational diabetes.<br>Pérez-Ferre et al. (2010) [21]		
**Domain**	**Review authors' judgement**	**Support to judgement**
**Random sequence generation**	High risk<br>Unclear<br>Low risk	?
**Allocation concealment**	High risk<br>Unclear<br>Low risk	?
**Blinding of participants and personnel**<br>Assessments should be made for each main outcome (or class of outcomes)	High risk<br>Unclear<br>Low risk	Patients weren't blinded: patients in the monitored group received a meter with a mobile phone. The mobile phone has a preinstalled application that allows the transmission of SBMG values to the central database through short message service.<br>Personnel: not mentioned if they were blinded. A diabetes team evaluated the patients data.
**Blinding of outcome assessment**<br>Assessments should be made for each main outcome (or class of outcomes)	High risk<br>Unclear<br>Low risk	?
**Incomplete outcome data**<br>Assessments should be made for each main outcome (or class of outcomes)	High risk<br>Unclear<br>Low risk	100 women gave their written informed consent and were allocated to:<br>- The intervention group (n = 50)<br>o 48 women completed the study<br>o 2 women were excluded; reason?<br>- The control group (n = 50)<br>o 49 women completed the study<br>o 1 woman was excluded; reason?<br>No re-inclusions were reported.
**Selective reporting**	High risk<br>Unclear<br>Low risk	Primary outcome: the assessment of the feasibility of a telemedicine system in the treatment of patients with gestational diabetes mellitus compared to traditional face-to-face visits.<br>Discussed outcomes:<br>- The use of the telemedicine system:<br>o Number of SMBG values<br>o Number of SMS<br>o Contain of the SMS<br>- Visits to the clinic<br>o Face to face visits<br>o Unscheduled visits<br>o Insulin-treated patients<br>o Gestational age at insulinitation<br>o Face-to-face visits<br>o Unscheduled visits
Overall outcome: of the 6 domains were 4 of high risk, 1 was unclear and 1 of low risk. The risk of		

bias is high.

**Article 9:** Impact of a Telemedicine System with Automated Reminders on Outcomes in Women with Gestational Diabetes Mellitus.<br>Homko et al. (2012) [19]		
**Domain**	**Review authors' judgement**	**Support to judgement**
**Random sequence generation**	High risk<br>Unclear<br>Low risk	?
**Allocation concealment**	High risk<br>Unclear<br>Low risk	?
**Blinding of participants and personnel**<br>Assessments should be made for each main outcome (or class of outcomes)	High risk<br>Unclear<br>Low risk	Patients and personnel weren't blinded: women in both groups were asked to monitor their blood glucose levels daily, perform fetal movement counting three times a day, and also record insulin doses and episodes of hypoglycemia. Women in the treatment group were asked to transmit this information either via the phone or Internet at least weekly to their healthcare providers. Women in the control group were asked to record this information in a logbook, which was reviewed by the medical team at prenatal visits.
**Blinding of outcome assessment**<br>Assessments should be made for each main outcome (or class of outcomes)	High risk<br>Unclear<br>Low risk	All data were analyzed based on subjects' assigned group regardless of their level of compliance with the testing protocol.
**Incomplete outcome data**<br>Assessments should be made for each main outcome (or class of outcomes)	High risk<br>Unclear<br>Low risk	80 women with gestational diabetes mellitus:<br>- 40 in the telemedicine group<br>o 3 women loss to follow-up<br>o 1 woman experienced a fetal loss at 19 weeks of gestation<br>- 40 in the control group<br>o 2 women loss to follow-up<br>No re-inclusions were reported.
**Selective reporting**	High risk<br>Unclear<br>Low risk	Primary outcomes: maternal glucose control, infant birth weight.<br>Secondary outcomes: pregnancy outcomes (mode of delivery, gestational age at delivery, neonatal intensive care unit admission, Apgar scores, rates of large for gestational age, and other neonatal morbidities) and system use.<br>Discussed outcomes:<br>- Fasting blood sugar<br>- Blood glucose (breakfast, lunch, dinner)<br>- Diabetes therapy:<br>o Diet<br>o Oral agents<br>o Insulin<br>- Maternal outcomes:

		o Caesarean delivery<br>o Pre-eclampsia/gestational hypertension<br>o Premature rupture of membranes<br>o Chorioamnionitis<br>- Neonatal outcomes:<br>o Birth weight<br>o Gestational age at delivery<br>o Apgar score: 1 min and 5 min<br>o Large for gestational age<br>o Neonatal hypoglycemia<br>o Intensive care unit admissions<br>o Preterm delivery<br>o Jaundice/hyperbilirubinemia<br>o Respiratory distress syndrome
Overall outcome: of the 6 domains were 4 of high risk and 2 of low risk. The risk of bias is high.		
