# Supplementary material for: Effectiveness of Telemonitoring in Obstetrics: Scoping Review
Source: J Med Internet Res. 2017 Sep 27;19(9):e327. doi: 10.2196/jmir.7266 (PMC5637065; doi:10.2196/jmir.7266)
Supplement: Multimedia Appendix 2 [file jmir_v19i9e327_app2.pdf]

Appendices 2: Summary table of included studies – maternal outcomes

Citation	Profile of included studies					Design of included studies							
	Nation-ality	Dates data collected	Study parti-cipants	Profes-sional feedback based on TM data	Data AT or MT	Design	Risk of bias	Size of experimental group	Size of control group	Study duration	Main types of data being transferred	Frequen-cy of data transmis sion
CHUMS Group (1995) [15]	United States of America	15/01/1991 – 27/05/1994	1292 singleton pregnancies at high risk for preterm labor	Yes	AT	MRCT	LR	N = 655	N = 637	From 24 – 32 weeks of gestation until 37 weeks of gestation or delivery	Uterine activity	Twice daily
Wapner et al. (1995) [25]	United States of America	02/1991 – 02/1993	218 singleton pregnancies at high risk for preterm labor or birth	Yes	AT	MRCT	LR	N = 107	N = 111	From 24 – 36 weeks of gestation until 37 weeks of gestation or delivery	Uterine activity	Twice daily
Corwin et al. (1996) [14]	United States of America	01/09/1988 – 31/08/1989	399 singleton pregnancies at high risk for preterm labor	Yes	AT	MRCT	LR	N = 174	N = 165	From 24 – 32 weeks of gestation until 37 weeks of gestation or delivery	Uterine activity	Twice daily
Brown et al. (1999) [22]	United States of	01/07/1991 – 01/01/1996	162 singleton pregnancie	Yes	AT	SRCT	MR	N = 82	N = 80	From 24 – 29 weeks of gestation	Uterine activity	Twice daily

	America		s at high risk for preterm labor							until delivery		
Morrison et al. (2001) [24]	United States of America	01/1992 – 11/1994	100 singleton pregnancies diagnosed with preterm labor	Yes	AT	RS	/	N = 60	N = 40	N/A	Uterine activity	N/A
Homko et al. (2007) [18]	United States of America	09/2004 – 05/2006	57 singleton pregnancies with GDM	Yes	MT	SRCT	HR	N = 32	N = 25	Less than 33 weeks' of gestation until delivery	Blood glucose levels, fetal movement counting's, insulin doses, episodes of glycaemia	Three times a week
Buysse et al. (2008) [23]	Belgium	01/01/2005 – 01/06/2006	456 episodes originating from 415 patients (patients are not further defined)	No	N/A	RS	/	N = 456	N/A	N/A	Costs	N/A
Dalfrà et al. (2009) [17]	Italy	N/A	276 pregnant women of	Yes	MT	MCRT	HRF	GDM N = 88 Diabetes type 1 N = 17	GDM N = 17 Diabets	GDM: a week after the	Capillary glucose data	Once a week and

			whom 240 diagnosed with GDM and 36 diagnosed with diabetes type 1						types 1 N = 15	diagnoses of GDM until delivery. Diabetes type 1: from first visit after conception until delivery.		more often is neces-sary
Pérez-Ferre et al. (2010) [19]	Spain	06/2007 – 12/2007	97 singleton pregnancie s diagnosed with GDM before 28 week of gestation	Yes	MT	SRCT	HR	N = 49	N = 48	From 24 – 32 weeks of gestation until delivery	Capillary glucose data	Once a week
Pérez-Ferre et al. (2010) [21]	Spain	06/2007 – 12/2007	97 singleton pregnancie s diagnosed with GDM before 28 week of gestation	Yes	MT	SRCT	HR	N = 49	N = 48	From 24 – 32 weeks of gestation until delivery	Capillary glucose data	Once a week
Rauf et al. (2011) [26]	United Kingdo m	01/01/2009 – 31/12/2010	70 women with healthy singleton pregnancie	Yes	AT	OS	/	N = 70	N/A	From 37 weeks of gestation until delivery	Uterine activity	Continu ously monitori ng from the

			s which had an induction of labour									moment of induction
Homko et al. (2012) [18]	United States of America	09/2007 – 11/2009	80 singleton pregnancies with GDM	Yes	MT	SRCT	HR	N = 40	N = 40	Less than 33 weeks' of gestation until delivery	Capillary glucose data	Four times a day
O'Brien et al. (2013) [27]	United Kingdom	N/A	15 singleton pregnancies which had an induction of labour	N/A	AT	QS	/	N = 15	N/A	From 37 weeks of gestation until delivery	Uterine activity	Continuously monitoring from the moment of induction
AT = automatically transferred; MT = manually transferred; MRCT = multicenter randomized controlled trials ; SRCT = single randomized controlled trials ; RS = retrospective study; OS = observational study; QS = qualitative study; LR = low risk; MR = medium risk; HR = high risk.<br>GDM = gestational diabetes mellitus; TM = telemonitoring group; CC: = control group.<br>N/A = not applicable												
