# Supplementary material for: Effectiveness of Telemonitoring in Obstetrics: Scoping Review
Source: J Med Internet Res. 2017 Sep 27;19(9):e327. doi: 10.2196/jmir.7266 (PMC5637065; doi:10.2196/jmir.7266)
Supplement: Multimedia Appendix 3 [file jmir_v19i9e327_app3.pdf]

Appendices 3: Summary table of included studies – neonatal outcomes

Citation	Profile of included studies					Design of included studies						
	**Nationality**	**Dates data collected**	**Study participants**	**Professional feedback based on TM data**	**Data AT or MT**	**Des-ign**	**Risk of bias**	**Size of experimental group**	**Size of control group**	**Study duration**	**Main types of data being transferred**	**Frequency of data transmission**
CHUMS Group (1995) [15]	United States of America	15/01/1991 – 27/05/1994	1292 singleton pregnancies at high risk for preterm labor	Yes	AT	MRCT	LR	N = 655	N = 637	From 24 – 32 weeks of gestation until 37 weeks of gestation or delivery	Uterine activity	Twice daily
Corwin et al. (1996) [14]	United States of America	01/09/1988 – 31/08/1989	399 singleton pregnancies at high risk for preterm labor	Yes	AT	MRCT	LR	N = 174	N = 165	From 24 – 32 weeks of gestation until 37 weeks of gestation or delivery	Uterine activity	Twice daily
Morrison et al. (2001) [24]	United States of America	01/1992 – 11/1994	100 singleton pregnancies diagnosed with preterm labor	Yes	AT	RS	/	N = 60	N = 40	N/A	Uterine activity	N/A
Homko et al. (2007) [18]	United States of	09/2004 – 05/2006	57 singleton pregnancie	Yes	MT	SRCT	HR	N = 32	N = 25	Less than 33 weeks' of gestation	Blood glucose levels, fetal	Three times a week

	America		s with GDM							until delivery	movement counting's, insulin doses, episodes of glycaemia	
Dalfrà et al. (2009) [17]	Italy	N/A	276 pregnant women of whom 240 diagnosed with GDM and 36 diagnosed with diabetes type 1	Yes	MT	MCRT	HR	GDM N = 88 Diabetes type 1 N = 17	GDM N = 17 Diabets types 1 N = 15	GDM: a week after the diagnoses of GDM until delivery. Diabetes type 1: from first visit after conception until delivery.	Capillary glucose data	Once a week and more often is neces-sary
Pérez-Ferre et al. (2010) [20]	Spain	06/2007 – 12/2007	97 singleton pregnancie s diagnosed with GDM before 28 week of gestation	Yes	MT	SRCT	HR	N = 49	N = 48	From 24 – 32 weeks of gestation until delivery	Capillary glucose data	Once a week
Pérez-Ferre et al. (2010) [21]	Spain	06/2007 – 12/2007	97 singleton pregnancie s diagnosed	Yes	MT	SRCT	HR	N = 49	N = 48	From 24 – 32 weeks of gestation until delivery	Capillary glucose data	Once a week

			with GDM before 28 week of gestation									
Homko et al. (2012) [18]	United States of America	09/2007 – 11/2009	80 singleton pregnancies with GDM	Yes	MT	SRCT	HR	N = 40	N = 40	Less than 33 weeks' of gestation until delivery	Capillary glucose data	Four times a day
Kuleva et al. (2012) [16]	France	1999 - 2010	singleton pregnancies complicated by fetal gastroschisis	Yes	N/A	RS	/	N = 97	N/A	From 24 – 32 weeks of gestation until 37 weeks of gestation or delivery	Fetal heart rate	Daily

AT = automatically transferred; MT = manually transferred; MRCT = multicenter randomized controlled trials ; SRCT = single randomized controlled trials ; RS = retrospective study; OS = observational study; QS = qualitative study; LR = low risk; MR = medium risk; HR = high risk.
GDM = gestational diabetes mellitus; TM = telemonitoring group; CC: = control group.
N/A = not applicable
